# Transient Receptor Potential Channel 4 Small-Molecule Inhibition Alleviates Migraine-Like Behavior in Mice

**DOI:** 10.3389/fnmol.2021.765181

**Published:** 2021-11-01

**Authors:** Cinder Faith Cohen, Arthur Silveira Prudente, Temugin Berta, Sang Hoon Lee

**Affiliations:** ^1^Pain Research Center, Department of Anesthesiology, University of Cincinnati Medical Center, Cincinnati, OH, United States; ^2^Neuroscience Graduate Program, University of Cincinnati College of Medicine, Cincinnati, OH, United States

**Keywords:** migraine, headache, peripheral sensory neurons, transient receptor potential channels, TRPC4, CGRP

## Abstract

Migraine is a common neurological disorder with few available treatment options. Recently, we have demonstrated the role of transient receptor potential cation channel subfamily C member 4 (TRPC4) in itch and the modulation of the calcitonin gene-related peptide (CGRP), a biomarker and emerging therapeutic target for migraine. In this study, we characterized the role of TRPC4 in pain and evaluated its inhibition as anti-migraine pain therapy in preclinical mouse models. First, we found that TRPC4 is highly expressed in trigeminal ganglia and its activation not only mediates itch but also pain. Second, we demonstrated that the small-molecule inhibitor ML204, a specific TRPC4 antagonist, significantly reduced episodic and chronic migraine-like behaviors in male and female mice after injection of nitroglycerin (NTG), a well-known migraine inducer in rodents and humans. Third, we found a significant decrease in CGRP protein levels in the plasma of both male and female mice treated with ML-204, which largely prevented the development of chronic migraine-like behavior. Using sensory neuron cultures, we confirmed that activation of TRPC4 elicited release of CGRP, which was significantly diminished by ML-204. Collectively, our findings identify TRPC4 in peripheral sensory neurons as a mediator of CGRP release and NTG-evoked migraine. Since a TRPC4 antagonist is already in clinical trials, we expect that this study will rapidly lead to novel and effective clinical treatments for migraineurs.

## Introduction

Migraine is a recurrent headache disorder affecting about 15% of the worldwide population with severe impact on patients’ lives, substantive healthcare costs, and loss of productivity (Vos et al., 2016). Migraine is characterized by various symptoms such as throbbing head pain, nausea, as well as hypersensitivity to light, sounds, and touch (Bigal et al., 2008; Goadsby, 2009). Hypersensitivity to touch (i.e., cutaneous mechanical allodynia) is often reported by migraineurs and is a common feature in animal models of migraine (Mathew et al., 2004; Pradhan et al., 2014; Lipton et al., 2017). While mechanisms leading to various symptoms are still incompletely known, transient receptor potential (TRP) channels are emerging as promising therapeutic targets for migraine (Dussor et al., 2014; Benemei and Dussor, 2019).

More than 30 TRP channels have been identified to date with a broad range of functions spanning from detection of various endogenous and exogenous ligands to sensing of temperature, pH, osmolarity, and mechanical stimuli (Clapham et al., 2001; Patapoutian et al., 2009). A number of these channels, including transient receptor potential cation channel subfamily A, member 1 (TRPA1); subfamily V, member 1 (TRPV1), subfamily V, member 4 (TRPV4), and subfamily M, member 8 (TRPM8) are highly expressed in primary sensory neurons and play a role in migraine (Dussor et al., 2014; Benemei and Dussor, 2019). Mutations in TRPA1, TRPV1, and TRPM8 have been linked to migraine (Chasman et al., 2011; Carreño et al., 2012; Ghosh et al., 2013; Dussor et al., 2014; Fan et al., 2014; Benemei and Dussor, 2019; Kowalska et al., 2020). Several studies have focused on a prominent role of TRPA1 in migraine, as a variety of environmental TRPA1 activators are well-known migraine triggers (S Benemei et al., 2014). Additionally, preclinical data support the activation of TRPA1 in migraine mechanisms such as increased dural blood flow and neurogenic vasodilatation *via* the release of calcitonin gene-related peptide (CGRP), a neuropeptide for which monoclonal antibodies have been recently FDA-approved for the treatment of migraine (Tso and Goadsby, 2017).

Transient receptor potential cation channel subfamily C members (TRPC1–TRPC7) are non-selective cation channels, which can mediate transmission of different forms of sensory information when activated by G protein-coupled receptors in various tissues (Sun et al., 2020). Among them, transient receptor potential cation channel subfamily C member 4 (TRPC4) and member 5 (TRPC5) are expressed in primary sensory neurons and have been implicated in itch and pain (Westlund et al., 2014; Wei et al., 2015; Lee et al., 2020; Sun et al., 2020; Sadler et al., 2021). We have recently characterized the expression of TRPC4 in primary sensory neurons expressing CGRP and demonstrated a functional and therapeutic role of TRPC4 in primary sensory neurons in an animal model of psoriasis (Lee et al., 2020). Importantly, we found that pharmacological inhibition of TRPC4 resulted in the reduction of psoriasiform skin inflammation and itch *via* a decrease of cutaneous pro-inflammatory cytokine and CGRP levels (Lee et al., 2020).

TRPC4 *via* its expression in primary sensory neurons and regulation of CGRP may represent a novel therapeutic target for migraine. However, the role of this channel in migraine has not yet been investigated. Here, we used a combination of behavioral, cellular, and biochemical approaches to reveal the function and therapeutic value of targeting TRPC4 in the NTG-induced animal model of migraine. Briefly, we demonstrated that TRPC4 is highly expressed in trigeminal sensory neurons mediating both itch and pain, and pharmacological inhibition of TRPC4 significantly reduced NTG-evoked cutaneous mechanical hypersensitivity in male and female mice, as well as high CGRP plasma levels associated with migraine.

## Materials and Methods

### Animals

Mouse experiments were initiated at 8–10 weeks of age in both male and female CD1 mice (strain code: 022, Charles River Laboratories, Wilmington, MA, United States). Mice were housed four per cage at 22 ± 0.5°C under a controlled 14/10 h light/dark cycle, with food and water available *ad libitum*. All experimental procedures were approved by the Institutional Animal Care and Use Committee at the University of Cincinnati, in accordance with the National Institute of Health and the International Association for the study of Pain. All results are reported according to Animal Research: reporting of *in vivo* Experiments (ARRIVE) guidelines (Kilkenny et al., 2010). Investigators were blind to animal treatments, no adverse effects were observed during these studies, and all animals were included in statistical analyses.

### Cheek Injection Mouse Model

The cheek injection model was utilized to measure pain and itch-related behaviors to different algogens and pruritogens, as previously reported (Shimada and LaMotte, 2008; Lee et al., 2018). Briefly, mouse cheeks were shaved under anesthesia, and mice were habituated to the testing plastic chambers (15 cm × 25 cm × 10 cm) daily for at least 2 days before testing. On the day of testing, mice were placed into the plastic chambers for 30 min and then removed for intradermal injection of 20 μL of the TRPC4 agonist Englerin A (100 μg, Cat# PHL82530, Millipore Sigma, St. Louis, MO, United States) with or without TRPC4 antagonist ML204 (40 μg, Cat# SML0400, Millipore Sigma). Injections of 20 μL of PBS were used as vehicle controls. After injections, mice were returned to the plastic chambers and video recorded for the quantification of itch- and pain-like behaviors. Specifically, we counted the time spent for pain-indicative wipes by a forearm and the number of bouts for itch-indicative scratches by a hind paw over 30 min.

### Immunofluorescence

Mice were anesthetized terminally with isoflurane and trigeminal ganglia (TG) tissues were removed and fixed in 4% paraformaldehyde overnight, and subsequently placed in a 30% sucrose solution for 24 h at 4°C. TG tissues were sectioned at 12 μm thickness using a cryostat and processed for immunofluorescence. Tissue sections were initially washed with PBS followed by incubation with BlockAid^TM^ blocking solution (Cat# B10710, Thermo Fisher Scientific) for 30 min. After blocking, tissue sections were incubated overnight with primary goat antibodies against CGRP (1:500; Cat#. ab36001, Abcam, Waltham, MA, United States), primary rabbit antibodies against GS (1:1,000; Cat#. Ab49873, Abcam), primary goat antibodies against IBA1 (1:500; Cat#. NB100-1028, Novus Biologicals, Centennial, CO, United States), and primary sheep antibodies against TRPC4 (1:1,000, Cat# OST00025W, Thermo Fisher Scientific). The following day, sections were washed with PBS then incubated for 1 h at room temperature with secondary anti-goat antibodies conjugated to Alexa Fluor 555 (1:500; Cat# A21432, Thermo Fisher Scientific), anti-sheep antibodies conjugated to Alex Fluor 488 (1:500; Cat# A11015, Thermo Fisher Scientific), and anti-rabbit antibodies conjugated to Alex Fluor 546 (1:500; Cat# A10040, Thermo Fisher Scientific). After incubation with secondary antibodies, some slides were incubated in 300 nM DAPI (Cat# D1306, Thermo Fisher Scientific) or fluorescent Nissl staining solution (1:100; Cat# N21483, Thermo Fisher Scientific) before being washed, air-dried, and coverslipped with Prolong Gold Antifade mounting medium (Thermo Fisher Scientific). Images were acquired and analyzed using a Keyence BZ-X800 microscope.

### DiI Labeling of Cutaneous Nerves

To determine whether TG neurons expressing TRPC4 innervate the cheek, we intradermally administered the well-known retrograde nerve tracer 1,10-dioctadecyl-3,3,30,30-tetramethylindocarbocyanine perchlorate (DiI, 100 μl of 1 mg/ml, Cat# D282, Thermo Fisher Scientific) into the cheek and collected TG tissues after 3 days. As expected, we observed intense red fluorescent signal in the neuronal cell bodies corresponding to the Ophthalmic (V1) and Maxillary (V2) TG branches, which mostly innervate the cheek.

### Nitroglycerin Mouse Model of Migraine

To produce an acute and chronic migraine animal model, mice were treated with nitroglycerin (NTG, Cat# 0517-4810-25, American Reagent, Shirley, NY, United States). Briefly, NTG was diluted on each test day in 0.9% saline solution to a concentration of 1 mg/ml for a dose of 10 mg/kg. Mice were intraperitoneally administered NTG once to mimic an acute migraine attack, or repeatedly every other day (day 0, 2, 4, 6, 8) to induce chronic migraine. A 0.9% saline solution was used as a vehicle control. The NTG model of migraine is now widely used and produces robust migraine-like pain behavior in mice (Pradhan et al., 2014). Migraine-like pain behavior was measured 2 h after NTG injection in the acute model, and on alternate days to NTG injections (days 1, 3, 5, 7, and 9) in the chronic model. On the final day of testing (Day 9), mice were anesthetized with isoflurane and blood was collected *via* intracardiac puncture, and lumbar DRG were harvested and placed in dry ice. Blood and tissue were stored in −20°C until further processing.

### Sensory Sensitivity Testing

Paw withdrawal thresholds to mechanical stimuli were evaluated using a series of calibrated von Frey monofilaments (Stoelting Co., Wood Dale, IL, United States). Briefly, mice were first acclimatized (60 min) in individual clear Plexiglas boxes on an elevated wire mesh platform to facilitate access to the plantar surface of the hind paws. Subsequently, a series of von Frey monofilaments (0.02, 0.07, 0.16, 0.4, 0.6, 1.0, and 1.4 g) were applied perpendicular to the plantar surface of the hindpaw. The test began with an application of a 0.6 g filament. A positive response was defined as a clear paw withdrawal or shaking. Whenever a positive response occurred, the next lower filament was applied, and whenever a negative response occurred, the next higher filament was applied. The testing consisted of six up-and-down stimuli, and the pattern of response was converted to a 50% von Frey withdrawal threshold as previously reported (Chaplan et al., 1994).

### Cell Cultures

Trigeminal ganglia and DRG cell cultures were prepared as previously described (Lee et al., 2018; Tonello et al., 2019). Briefly, mice were terminally anesthetized with isoflurane, and TGs or lumbar, thoracic, and cervical DRGs were bilaterally removed aseptically from 8-week-old mice. Removed TG or DRG tissues were incubated in papain (60 U, Cat# P3125, Millipore Sigma) for 20 min at 37°C followed by collagenase (1 mg/ml, Cat# C6885, Millipore Sigma) for another 20 min at 37°C. Tissues were mechanically dissociated, and cells were then cultured in Dulbecco’s Minimal Essential Medium (Cat# 15017CV, Corning Inc., Corning, NY, United States) completed with 10% fetal bovine serum and 1% pen/strep in eight-well chambered cell culture slides (Cat# 354118, Corning) precoated with a cell and tissue adhesive (Geltrex, Cat# A1569601, Thermo Fisher Scientific). Cultures were incubated at least 24 h at 37°C with 5% carbon dioxide before each experiment. These cultures were treated with PBS vehicle control or TRPC4 agonist Englerin A (100 nM to 10 mM) with or without ML204 (100 μM) for 24 h and then culture media were collected and stored at −80°C for further ELISA analyses.

### ELISA

An ELISA kit for CGRP was purchased from Cayman Chemical (Cat# 589001, Ann Arbor, MI, United States). ELISA was performed using culture media or protein extracts from DRG tissues. For protein extracts, mice were terminally anesthetized with isoflurane 9 days after repeated NTG or vehicle injections and lumbar DRGs were rapidly removed. DRGs were homogenized in RIPA lysis and extraction buffer (Cat# 89900, Thermo Fisher Scientific, Waltham, MA, United States), and protein concentrations were measured by the Qubit protein assay (Cat# Q33211, Thermo Fisher Scientific). A standard curve was included in each experiment, and CGRP protein levels measured according to the manufacturer’s instruction using a PerkinElmer EnVision plate reader.

### Quantitative Real-Time RT-PCR

Mice were terminally anesthetized with isoflurane and TGs from naïve mice or lumbar DRGs from mice repeatedly injected with NTG or vehicle over 9 days were rapidly removed and stored in −20°C until further processing. TGs or DRGs were mechanically dissociated with a grinder, and total RNA was extracted using TRIzol RNA isolation reagent (Cat# 15596026, Thermo Fisher Scientific) and Direct-zol RNA MiniPrep kit (Zymo Research, Irvine, CA, United States). The amount and quality of RNA were assessed by SimpliNano UV-Vis Spectrophotometer (General Electric, Boston, MA, United States) and then converted into cDNA using a high-capacity cDNA reverse transcription kit (Cat# 4368814, Thermo Fisher Scientific). Specific primers for TRPCs, neuropeptides, and glyceraldehyde 3-phosphate dehydrogenase (GAPDH) were obtained from PrimerBank (Wang et al., 2012). Primer sequences are shown in Supplementary Table 1. Quantitative real-time RT-PCR (qPCR) was performed on a QuantStudio 3 Real-Time PCR System (Thermo Fisher Scientific) using PowerUp SYBR Green Master Mix (Cat# A25741, Thermo Fisher Scientific). All samples were analyzed at least in duplicate and normalized by GAPDH expression. The relative expression ratio per condition was calculated based on the method described by Pfaffl (2001).

### Statistical Analysis

Prism 9.0 (GraphPad, San Diego, CA, United States) was used for statistical analysis. Statistical tests used are specified at the end of each figure legend. Generally, unpaired, two-tailed Student’s *t*-test was used for analyses between two groups, whereas one-way or two-way ANOVA coupled with a specific multiple-comparisons test was used for multiple groups and/or conditions. Sample sizes were designed to generate 80% power at two-sided *P* < 0.05. Data are presented as mean ± SEM, with value derived from independent biological replicates. Adobe Illustrator 25.0 (Adobe, San Jose, CA, United States) was used for illustrations and figure organization.

## Results

### TRPC4 Mediates Itch and Pain

**FIGURE 1:**
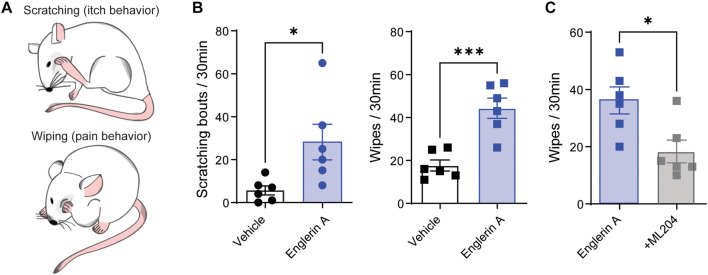
TRPC4 activation induces both pain and itch. **(A)** Illustration of cheek injection model by which pruritogens elicit hind paw scratching responses and algogens evoke unilateral front paw wiping responses. **(B)** Treatment with Englerin A (100 μg), a TRPC4 agonist, evokes significant increases in both scratching and wiping bouts in male mice. **(C)** ML204 (40 μg), a TRPC4 antagonist, significantly attenuates Englerin A-evoked wipes. ^∗^*p* < 0.05, ^∗∗∗^*p* < 0.001, vs. vehicle (PBS). Two-tailed, unpaired Student’s *t*-test, *n* = 6 mice/group.

We have recently reported the involvement of TRPC4 in acute and chronic itch (Lee et al., 2018, 2020). However, there is evidence of an involvement of TRPC4 in visceral and neuropathic pain (Westlund et al., 2014; Wei et al., 2015). To test whether TRPC4 is involved in both itch and pain, we used the cheek mouse model (Shimada and LaMotte, 2008) and examined whether intradermal injection of the TRPC4 agonist Englerin A (20 μl, 100 μg) evokes itch-indicative scratches and pain-indicative wipes (Figure 1A). As expected, injection of Englerin A elicited acute itch-like behaviors demonstrated by the number of scratching bouts directed to the site of injection (Figure 1B). However, Englerin A also induced pain-like behaviors as quantified by an increase in time spent wiping the injected site (Figure 1B). Because Englerin A has been reported to potentially activate TRPC5 (Akbulut et al., 2015), too, we administered ML204 (40 μg), a specific TRPC4 antagonist, and observed that it significantly reduced the duration of wipes (Figure 1C), suggesting that activation of TRPC4 is involved in pain.

### Characterization of TRPC4 Expression in Trigeminal Ganglia

**FIGURE 2:**
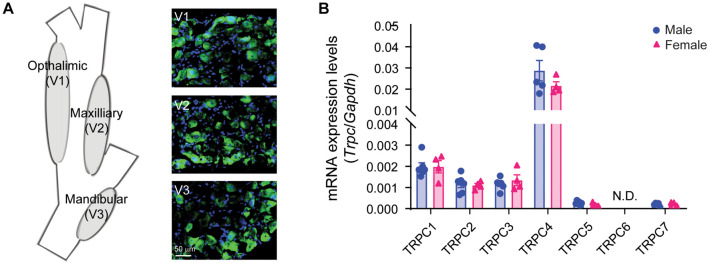
Characterization of TRPC4 expression in trigeminal ganglia (TG) tissue. **(A)** Illustration of the TG and its three branches (V1–V3) innervating the head and face, and TRPC4 immunofluorescence (green) in the V1, V2, and V3 areas. DAPI (blue) was used as tissue counterstaining. **(B)** Transcriptional analysis of the TRPCs in TG tissues from male and female mice. GAPDH was used as the housekeeping gene. *n* = 4–6 mice/sex/group.

The cheek is innervated by the sensory neurons residing in the trigeminal ganglia (TG). Previously, we found that TRPC4 protein is colocalized with CGRP-positive neurons in dorsal root ganglia (DRG) (Lee et al., 2020). Immunofluorescence revealed robust expression of TRPC4 protein in all three branches of the TG, with the maxillary branch exhibiting 30.9% ± 3.5 S.D. of TRPC4 positive neurons (Figure 2A). Similar to our previous studies in DRG tissues, TRPC4 was found uniquely in neurons and in particular CGRP-positive TG neurons innervating the skin (Supplementary Figures 1A,B). Transcriptional analysis revealed that all TRPC channels were expressed in the TG except for *Trpc6*, which was undetectable (Figure 2B). Notably, we found similar expression in male and female mice with high expression of *Trpc4*, moderate expression of *Trpc1*, *Trpc2*, and *Trpc3*, and minimal expression of *Trpc5* and *Trpc7*. This is in contrast with what we found in DRG, where *Trpc3* was the highest expressed gene, whereas *Trpc4* was only moderately expressed. These data suggest a major role for TRPC4 expression in TG and a potential function in TG-driven pain.

### TRPC4 Antagonist ML204 Attenuates NTG-Induced Mechanical Hypersensitivity

**FIGURE 3:**
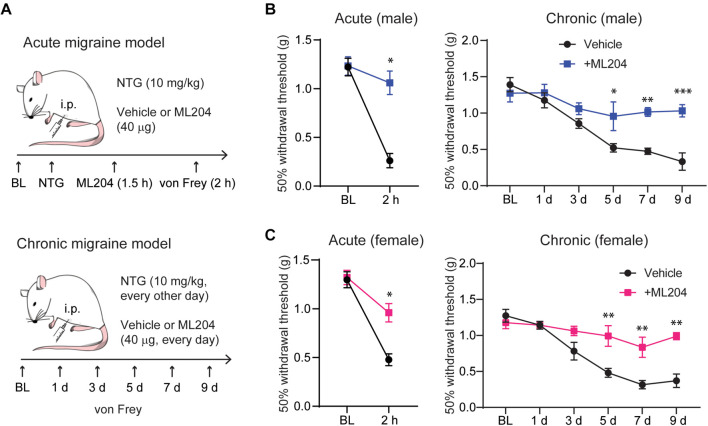
TRPC4 inhibitor ML204 alleviates NTG-induced episodic and chronic pain in male and female mice. **(A)** Illustration of the mouse models used for acute (episodic) and chronic migraine, including timelines for treatment and behavioral tests. TRPC4 inhibitor ML204 prevents both acute and chronic mechanical hypersensitivity in male **(B)** and female **(C)** mice, which was assessed using a series of von Frey filaments applied to the plantar surface of the hind paw. ^∗^*p* < 0.05, ^∗∗^*p* < 0.01, ^∗∗∗^*p* < 0.001, vs. vehicle. Two-way ANOVA followed by Bonferroni’s *post hoc* test, *n* = 5–6 mice/sex/group.

Because TG and CGRP are critical in migraine pathophysiology and considering our finding that TRPC4 is highly expressed in TG tissues and its inhibition reduced CGRP in a model of psoriasis (Lee et al., 2020), we asked if TRPC4 inhibition may offer a therapeutic option for the treatment of migraine. We used two mouse models of migraine (Figure 3A) consisting of: (1) a single intraperitoneal injection of NTG (i.p., 10 mg/kg) mimicking an acute episodic migraine attack and (2) repeated i.p. injection of NTG (10 mg/kg, one injection every other day) mimicking chronic migraine (Pradhan et al., 2014). Both models evoke the development of a systemic migraine-like mechanical hypersensitivity that can be tested using von Frey monofilaments applied to the hindpaw (Pradhan et al., 2014). We found that a single i.p. injection of the TRPC4 small-molecule inhibitor ML204 (40 μg) significantly reversed the acute mechanical hypersensitivity, as well as daily i.p. injections of ML204 (40 μg, every day) significantly prevented the development of chronic mechanical hypersensitivity in male mice (Figure 3B). It is well documented that women are more susceptible to migraine and the underlying mechanisms in humans and rodents may differ between the two sexes (James et al., 2018; Avona et al., 2019). Remarkably, male and female mice display similar responses to treatments with ML204 significantly reversing acute-induced mechanical hypersensitivity as well as preventing chronic-evoked mechanical hypersensitivity in female mice (Figure 3C). This suggests that TRPC4 inhibition can potentially be used as anti-migraine pain therapy in both men and women.

### ML204 Reduced CGRP Transcripts in DRG Tissues and Protein Levels in Blood Plasma

**FIGURE 4:**
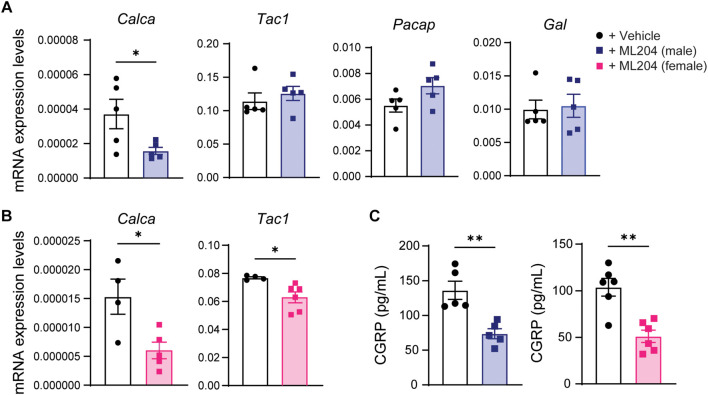
TRPC4 inhibitor ML204 decreases CGRP transcriptional and protein levels in the chronic migraine mouse model **(A,B)** Transcriptional analyses of various neuropeptides in DRG tissues from male **(A)** and female **(B)** mice treated with ML204 or vehicle control (PBS) after 9 days in the NTG-evoked chronic pain model. ^∗^*p* < 0.05 vs. vehicle, two-tailed, unpaired Student’s *t*-test, *n* = 4–6 mice/sex/group. **(C)** Plasma levels of CGRP protein in male and female mice treated with ML204 or vehicle control (PBS) after 9 days in the NTG-evoked chronic pain model. ^∗∗^*p* < 0.01, vs. vehicle, two-tailed, unpaired Student’s *t*-test, *n* = 5–6 mice/sex/group.

To explore the mechanisms by which daily TRPC4 inhibition attenuates pain hypersensitivity, we examined transcriptional changes in several neuropeptides enriched in primary sensory neurons and/or previously associated with migraine (Tajti et al., 2015). We found that male mice treated with ML204 vs. vehicle exhibited a significant decrease in transcripts for CGRP (*Calca*) and no other neuropeptides such as the pituitary adenylate cyclase-activating polypeptide (*Pacap*) or precursor of substance P (*Tac1*) in DRG tissues (Figure 4A and Supplementary Figure 2A). Similarly, female mice showed a significant decrease in transcripts for CGRP but also substance P (Figure 4B), whereas other neuropeptides were unchanged (Supplementary Figure 2B). We confirmed a systemic regulation of CGRP at the protein level and found a significant reduction of CGRP protein in blood plasma in both male and female mice after daily treatment with ML204 compared to vehicle (Figure 4C). Of note, CGRP plasma levels were also found significantly elevated in migraineurs and may represent a biomarker of migraine (Cernuda-Morollón et al., 2013).

### TRPC4 Mediates CGRP Release in Cultured Primary Sensory Neurons

**FIGURE 5:**
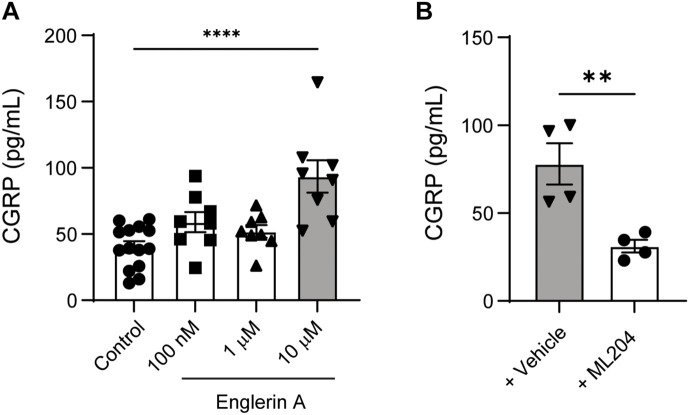
TRPC4 activity controls CGRP release in cultured peripheral sensory neurons. **(A)** Englerin A (10 μM) elicited CGRP release in the culture media of DRG neurons. ^****^p < 0.0001, two-tailed, unpaired Student’s *t*-test, *n* = 8–14 wells/group. **(B)** ML204 (100 μM) significantly reduced Englerin A-evoked CGRP release in the culture media. **p < 0.01, two-tailed, unpaired Student’s *t*-test, *n* = 4 wells/group.

Having demonstrated that systemic delivery of a TRPC4 antagonist, ML204, results in reduced pain and CGRP levels, we next sought to examine the direct effects of a TRPC4 agonist (Englerin A) and antagonist (ML204) on cultured primary sensory neurons. DRG cultured neurons treated with Englerin A exhibited a dose-dependent increase in CGRP release, with 10 μM evoking a robust increase in CGRP protein levels in the culture medium (Figure 5A). In line with a specific action on TRPC4, ML204 treatment resulted in a significant reduction of CGRP release evoked by Englerin A (Figure 5B). Because we needed larger amounts of cultured neurons for our *in vivo* pharmacological approaches, we have run our experiments collecting neurons from 30 to 40 DRG tissues vs. two TG tissues per animal. However, we confirmed a significant release of CGRP in TG cultured neurons after incubation with Englerin A (Supplementary Figure 3). These results indicate a direct effect of TRPC4 activation on CGRP release in primary sensory neurons.

## Discussion

Several members of the TRP channel family play a pivotal role in the generation of various pain conditions, including migraine (Benemei and Dussor, 2019). Here, we have shown that TRPC4 is highly expressed in cutaneous peptidergic sensory neurons in the trigeminal ganglia, a key structure implicated in the generation of migraine (Goadsby, 2009). Indeed, we further demonstrated that pharmacological inhibition of TRPC4 significantly prevented NTG-evoked cutaneous mechanical hypersensitivity and increased plasma levels of migraine-linked neuropeptide CGRP.

A growing number of studies have linked TRP channels with migraine, specifically TRPA1 and TRPV1 channels which are expressed in trigeminal sensory neurons (Patapoutian et al., 2009; Dussor et al., 2014). We have previously identified TRPC4 expression in sensory neurons from dorsal root ganglia as a major driver of serotonergic and psoriasiform itch (Lee et al., 2018, 2020). However, other studies have shown an additional role of TRPC4 in visceral and neuropathic pain (Westlund et al., 2014; Wei et al., 2015). To test whether TRPC4 is associated with itch and pain, we used intradermal cheek injections of the TRPC4 agonist Englerin A and then characterized pruriceptive function by quantifying ensuing itch behavioral responses (hindlimb scratching of cheek) or pain (forelimb wiping of cheek) (Shimada and LaMotte, 2008; Lee et al., 2018). Although activation of TRPC4 in association with serotonin receptor 2B led to intense itch but no pain behavior (Lee et al., 2018), we found that direct stimulation of TRPC4 induced both itch and pain behaviors.

Activation of TRPC4 in primary sensory neurons is likely responsible for these behaviors since we have reported that about 35% of all cultured primary sensory neurons displayed calcium influx after direct stimulation with Englerin A (Lee et al., 2020). To note, calcium influx was almost or totally abolished in neurons from mice lacking TRPC4 expression, suggesting TRPC4 as a major driver of Englerin A in primary sensory neurons. However, we can’t exclude residual actions of Englerin A through TRPC5 (Akbulut et al., 2015). In line with our calcium analysis, we found that about 31% of trigeminal sensory neurons expressed TRPC4 protein with most of them also expressing CGRP and innervating the skin. In contrast, we found no expression of TRPC4 in non-neuronal cells in TG tissue such as satellite glial cells and macrophages. Similar to what we reported for dorsal root ganglia, transcriptional analyses revealed the presence of most TRPC channels in trigeminal ganglia of male and female mice. However, TRPC4 expression was the highest in trigeminal ganglia, whereas TRPC3 expression was the highest in dorsal root ganglia. This is not surprising as differences in transcriptome and translatome have already been documented between these two tissues (Megat et al., 2019). Because of the high expression of TRPC4 in trigeminal ganglia and colocalization with CGRP, we hypothesized that TRPC4 may play a role in migraine.

Drugs targeting TRP channels have been explored for the relief of various pain conditions, but their inhibition has been associated with undesirable adverse effects such as hyperthermia evoked by TRPV1 antagonists (Patapoutian et al., 2009). TRPC4 is not associated with thermoregulation and may represent a better therapeutic target. To test whether TRPC4 inhibition can achieve analgesic effect in migraine, we used systemic injections of NTG in mice. NTG is commonly used to evoke migraine in humans, and in rodents, NTG elicits mechanical hypersensitivity that mimics the sensory hypersensitivity associated with migraine (Mathew et al., 2004; Pradhan et al., 2014; Lipton et al., 2017). Furthermore, migraine often begins as an episodic disorder but can progress to a chronic disorder. Therefore, we tested the effect of TRPC4 inhibition in models of episodic migraine by a single injection of NTG and chronic migraine by repeated injections of NTG (Pradhan et al., 2014). We found that the TRPC4 specific inhibitor ML204 reduced cutaneous mechanical hypersensitivity after a single injection and prevented the development of this hypersensitivity after repeated injections. In addition, women are more susceptible to migraine (James et al., 2018) and different underlying mechanisms have been identified between sexes in mice (Avona et al., 2019), but our results show similar reduction and prevention of mechanical hypersensitivity between male and female mice.

Several neuropeptides can be released from sensory neurons and have been reported to play a critical role in migraine (Tajti et al., 2015). In this study, we have assessed the transcriptional regulation of various neuropeptides in DRG tissues from male and female mice after daily treatment with ML204 and observed a transcriptional decrease only for CGRP transcripts in male mice and CGRP and substance P in female mice. CGRP plasma levels were also reduced in both male and female mice after daily treatment with ML204 suggesting a systemic regulation of this protein. Increases in CGRP levels were observed not only in plasma but also in saliva and CSF of migraineurs during an attack (Edvinsson and Goadsby, 1994; Jang et al., 2011). Previously, we have reported that the therapeutic effect of TRPC4 may be mediated by inhibition of CGRP release from peripheral sensory neurons that innervate various tissues (Lee et al., 2020). Consistent with this report, we demonstrated that TRPC4 activation by Englerin A in cultured peripheral sensory neurons resulted in the release of CGRP, which was inhibited by ML204. Although Englerin A may also activate TRPC5 (Akbulut et al., 2015; Carson et al., 2015), we did not observe an inhibition of CGRP release by a TRPC5 antagonist (preliminary data not shown). CGRP has been implicated in migraine for decades and monoclonal antibodies that target peripheral CGRP signaling are emerging as novel therapeutics (Russell et al., 2014; Tso and Goadsby, 2017). While it has been shown that estrogen can regulate the release of CGRP (Stucky et al., 2011; Pota et al., 2017), we did not find any sex differences in the analgesic effect of TRPC4 and its regulation of CGRP plasma levels. Recently, it has been reported that CGRP may have female-specific actions at nerve endings in peripheral tissues, such as the dura (Avona et al., 2019). However, the small-molecule inhibitor ML204 may penetrate different nervous system tissues and act at nerve endings in peripheral tissues as well as in central nervous system tissues. Furthermore, ML204 may target the release of CGRP in the central nervous system, such as in the brainstem, which may not present sexual dimorphism. It should be noted that even though CGRP is highly expressed in rodent and human fibers projecting to the brainstem (Eftekhari and Edvinsson, 2011), which is active and plays a role in migraine pathophysiology, no study has evaluated the role of male vs. female brainstem in migraine (Maleki and Androulakis, 2019). It is also important to consider that ML204 may regulate not only CGRP but also other neuropeptides. For instance, we found that ML204 treatment slightly but significantly decreased transcripts for substance P in female mice, suggesting that future studies should expand our investigation to additional neuropeptides and molecules associated with migraine.

This investigation presents other limitations that warrant further studies. While hypersensitivity to touch is present in many migraineurs and cutaneous mechanical allodynia is well characterized in the NTG animal model of migraine (Mathew et al., 2004; Pradhan et al., 2014; Lipton et al., 2017), additional animal models and symptoms associated with migraine should be explored to validate the inhibition of TRPC4 as a novel therapeutic approach. TRPC4 inhibition can occur in the peripheral and central nervous systems. Although we showed data suggesting that TRPC4 regulated CGRP release in peripheral sensory neurons, it has been reported that amygdaloid TRPC4/C5 contributes to the maintenance of pain hypersensitivity and pain affect in an animal model of neuropathic pain (Wei et al., 2015). The activation and role of amygdaloid TRPC4/TRPC5 in migraine should be further explored in future studies. Activation of TRPC5 by endogenous lysophosphatidylcholine in peripheral sensory neurons has recently emerged as a mediator of inflammatory and neuropathic pain (Sadler et al., 2021). Interestingly, lysophosphatidylcholine is increased in various animal pain models and its application to meningeal tissue elicited migraine-like behavior. In this study, we did not identify the endogenous agonists of TRPC4 that may be associated with migraine. However, we have previously shown that TRPC4 is associated with serotonergic signaling (Lee et al., 2018, 2020) in peptidergic neurons and various studies have implicated serotonin in the pathogenesis of migraine (Aggarwal et al., 2012). Further investigations should be carried out to define the endogenous agonists of TRPC4, as well as its expression with serotonergic receptors in rodent and human tissues. Of note, a discrepancy between mouse and human TRPC5 expression in peripheral sensory neurons has been reported (Sadler et al., 2021), and thus a precise characterization of TRPC4 expression in human tissues will be crucial for the evaluation of the translational potential of TRPC4 as a therapeutic target.

In summary, here we identify a high expression of TRPC4 in trigeminal ganglia and its implication in itch and pain. In particular, we have demonstrated that TRPC4 inhibition can alleviate episodic and chronic migraine-like behavior in male and female mice treated with NTG. Mechanistically, we also showed that this inhibition resulted in reductions in plasma levels of CGRP and release of CGRP in cultured peripheral sensory neurons. Targeting TRP channels holds great promise for pain treatment, and we anticipate that strategies targeting TRPC4 in peripheral sensory neurons will be beneficial in treating migraine.

## Data Availability Statement

The raw data supporting the conclusions of this article will be made available by the authors, without undue reservation.

## Ethics Statement

The animal study was reviewed and approved by the Institutional Animal Care and Use Committee at the University of Cincinnati.

## Author Contributions

CC, TB, and SL designed the study and wrote the manuscript. CC, AP, and SL conducted the experiments and acquired and analyzed the data. TB and SL supervised the study. All authors revised the manuscript and approved the submitted version.

## Conflict of Interest

The authors declare that the research was conducted in the absence of any commercial or financial relationships that could be construed as a potential conflict of interest.

## Publisher’s Note

All claims expressed in this article are solely those of the authors and do not necessarily represent those of their affiliated organizations, or those of the publisher, the editors and the reviewers. Any product that may be evaluated in this article, or claim that may be made by its manufacturer, is not guaranteed or endorsed by the publisher.
