# Supplementary material for: Transient Receptor Potential Channel 4 Small-Molecule Inhibition Alleviates Migraine-Like Behavior in Mice
Source: Front Mol Neurosci. 2021 Nov 1;14:765181. doi: 10.3389/fnmol.2021.765181 (PMC8591066; doi:10.3389/fnmol.2021.765181)
Supplement: Supplementary file 1 [file Data_Sheet_1.pdf]

frontiers

Supplementary Material

Table

Supplementary Table 1: Quantitative Real-Time RT-PCR Primer Sequences

Gene	Forward Primer (5'-3')	Reverse Primer (5'-3')	Accession Number
**Trpc1**	TACGGTTGTCAGTCCGCAGA	TCGTTTTGGCCGATGATTAAGTA	NM_011643.4
**Trpc2**	GCCATGTGGTGTCATTTTCCT	GTTGTCCAGTCTTGTCCTGAG	NM_011644.3
**Trpc3**	TCGAGAGGCCACACGACTA	CTGGACAGCGACAAGTATGC	NM_019510.2
**Trpc4**	TGTATCTGGCAACAATCTCCTTG	CATGTCCCATGATTCCCGTGG	NM_016984.3
**Trpc5**	GTGTATCCAGTTCGGAGGTAGA	CCTCGCTTGATAAGGCAATGA	NM_009428.3
**Trpc6**	AGCCAGGACTATTTGCTGATGG	AACCTTCTTCCCTTCTCACGA	NM_013838.2
**Trpc7**	CTTCCTGGACTCGGCTGAGTA	GCGTTCTGCCCCATGTAGT	NM_012035.3
**Calca**	GAGGGCTCTAGCTTGGACAG	AAGGTGTGAAACTTGTTGAGGT	NM_007587.2
**Gal**	GGCAGCGTTATCCTGCTAGG	CTGTTCAGGGTCCAACCTCT	NM_010253.4
**Npy**	ATGCTAGGTAACAAGCGAATGG	TGTCGCAGAGCGGAGTAGTAT	NM_023456.3
**Pacap**	ACCATGTGTAGCGGAGCAAG	CTGGTCGTAAGCCTCGTCT	NM_009625.3
**Sst**	ACCGGGAAACAGGAACTGG	TTGCTGGGTTCGAGTTGGC	NM_009215.1
**Tac1**	ATTCCTTTGTTGGACTAATGGGC	ACGTCTTCTTTCGTAGTTCTGC	NM_009311.3
**Gapdh**	AGGTCGGTGTGAACGGATTTG	GGGGTCGTTGATGGCAACA	NM_001289726.1

Figures

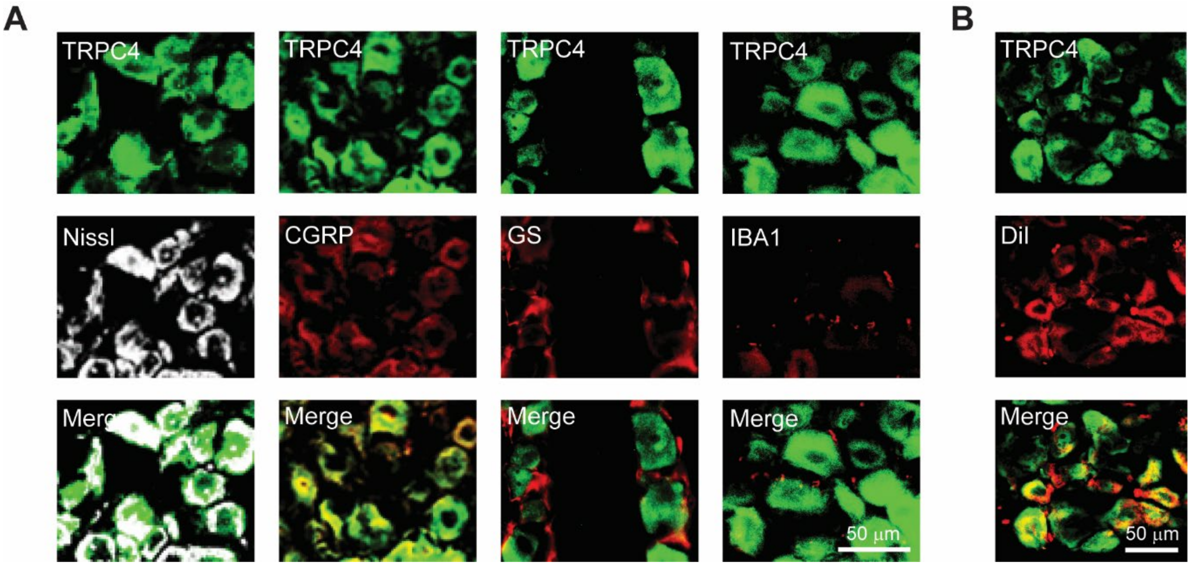

Supplementary Figure 1: Expression of TRPC4 in TG tissue. (A) Representative immunofluorescence images of TRPC4 protein expression in TG neurons, identified by Nissl counterstaining and CGRP staining. To note, TRPC4 is not expressed in satellite glial cells and macrophages stained respectively with well-known markers glutamine synthetase (GS) and ionized calcium-binding adapter molecule 1 (IBA1). **(B)** Representative immunofluorescence images of TRPC4 protein expression in cutaneous TG neurons, identified by injection of the retrograde tracing dye DiI into the cheek.

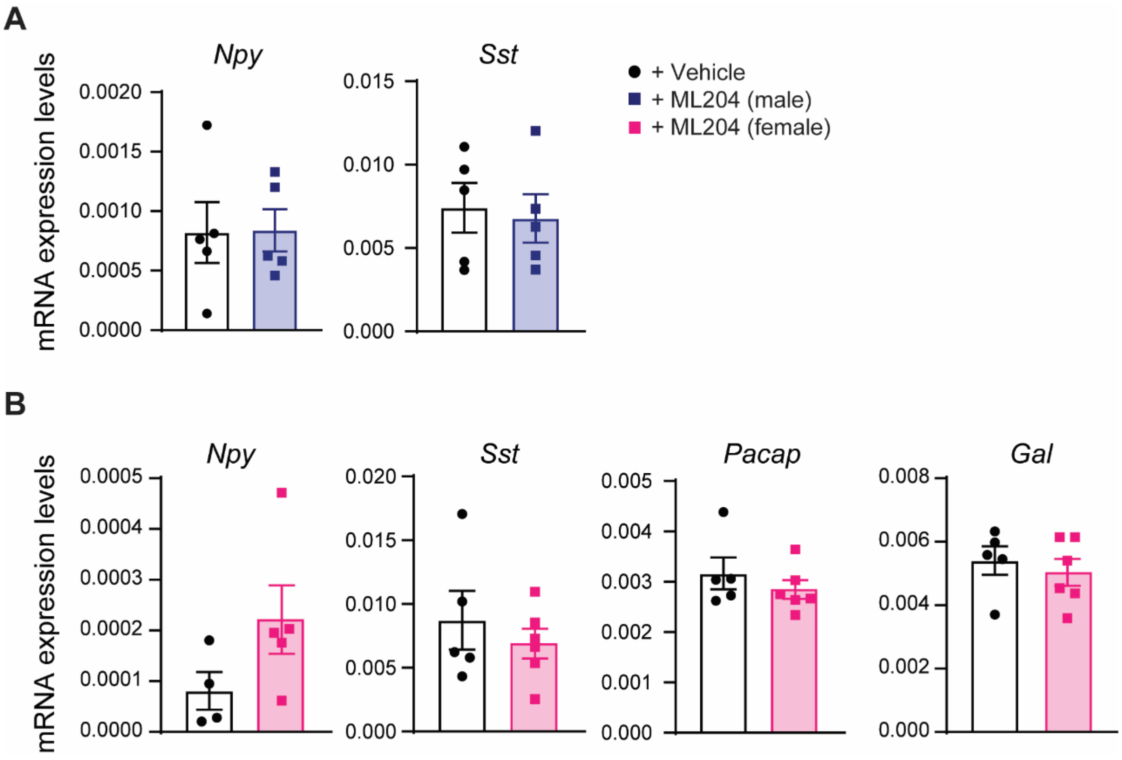

Supplementary Figure 2: **Transcriptional analyses of various neuropeptides in DRG tissues.** Samples were collected from male and female mice treated with ML204 or vehicle control (PBS) after 9 days in the NTG-evoked chronic pain model. n = 4-6 mice/sex/group.

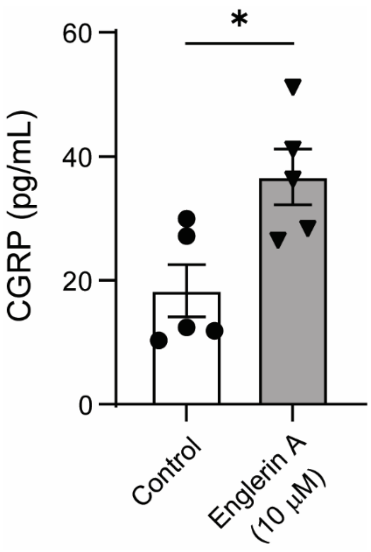

Supplementary Figure 3: TRPC4 activity controls CGRP release in cultured TG neurons. Englerin A (10 µM) elicits CGRP release in the culture media of cultured neurons. *$p < 0.05$, Two-tailed, unpaired Student's t-test, n = 5 wells/group.
